# Polydopamine nanoparticle-mediated mild photothermal therapy for inhibiting atherosclerotic plaque progression by regulating lipid metabolism of foam cells

**DOI:** 10.1093/rb/rbad031

**Published:** 2023-03-25

**Authors:** Shuangshuang Tu, Wenzhi Ren, Jinru Han, Haijing Cui, Ting Dai, Haoxuan Lu, Yanqing Xie, Wenming He, Aiguo Wu

**Affiliations:** Department of Cardiology, The First Affiliated Hospital of Ningbo University, Ningbo, Zhejiang Province 315020, China; Cixi Institute of Biomedical Engineering, International Cooperation Base of Biomedical Materials Technology and Application, Chinese Academy of Science (CAS) Key Laboratory of Magnetic Materials and Devices & Zhejiang Engineering Research Center for Biomedical Materials, Ningbo Institute of Materials Technology and Engineering CAS, Ningbo 315201, China; Cixi Institute of Biomedical Engineering, International Cooperation Base of Biomedical Materials Technology and Application, Chinese Academy of Science (CAS) Key Laboratory of Magnetic Materials and Devices & Zhejiang Engineering Research Center for Biomedical Materials, Ningbo Institute of Materials Technology and Engineering CAS, Ningbo 315201, China; Advanced Energy Science and Technology Guangdong Laboratory, Huizhou 516000, China; Cixi Institute of Biomedical Engineering, International Cooperation Base of Biomedical Materials Technology and Application, Chinese Academy of Science (CAS) Key Laboratory of Magnetic Materials and Devices & Zhejiang Engineering Research Center for Biomedical Materials, Ningbo Institute of Materials Technology and Engineering CAS, Ningbo 315201, China; University of Chinese Academy of Sciences, Beijing 101408, China; Cixi Institute of Biomedical Engineering, International Cooperation Base of Biomedical Materials Technology and Application, Chinese Academy of Science (CAS) Key Laboratory of Magnetic Materials and Devices & Zhejiang Engineering Research Center for Biomedical Materials, Ningbo Institute of Materials Technology and Engineering CAS, Ningbo 315201, China; Department of Cardiology, The First Affiliated Hospital of Ningbo University, Ningbo, Zhejiang Province 315020, China; Department of Cardiology, The First Affiliated Hospital of Ningbo University, Ningbo, Zhejiang Province 315020, China; Department of Cardiology, The First Affiliated Hospital of Ningbo University, Ningbo, Zhejiang Province 315020, China; Department of Cardiology, The First Affiliated Hospital of Ningbo University, Ningbo, Zhejiang Province 315020, China; Cixi Institute of Biomedical Engineering, International Cooperation Base of Biomedical Materials Technology and Application, Chinese Academy of Science (CAS) Key Laboratory of Magnetic Materials and Devices & Zhejiang Engineering Research Center for Biomedical Materials, Ningbo Institute of Materials Technology and Engineering CAS, Ningbo 315201, China; Advanced Energy Science and Technology Guangdong Laboratory, Huizhou 516000, China

**Keywords:** polydopamine nanoparticles, photothermal therapy, atherosclerosis, lipid metabolism

## Abstract

Since apoptosis of foam, cells can induce plaque instability, reducing intracellular lipid content while protecting foam cells from apoptosis is beneficial for the safe and efficient therapy of atherosclerosis. In this study, osteopontin-coupled polydopamine (PDA-OPN) nanoparticles were synthesized and applied to target mild photothermal therapy (PTT) of atherosclerosis. The results from laser confocal microscopy indicate that PDA-OPN nanoparticles can be specially recognized and absorbed by foam cells. Under near-infrared laser irradiation, the mild photothermal generated by PDA-OPN decreases intracellular lipid accumulation but does not induce cell apoptosis. *In vivo* treatments demonstrate that mild PTT can substantially reduce plaque area and improve plaque stability by upregulating the expression of plaque fibrosis in ApoE^−/−^ mice. Our findings reinforce that the PDA-OPN nanoparticle-mediated mild PTT can inhibit atherosclerotic progression, which provides new insights for developing safe and effective treatment methods for atherosclerosis.

## Graphical Abstract

**Figure:**
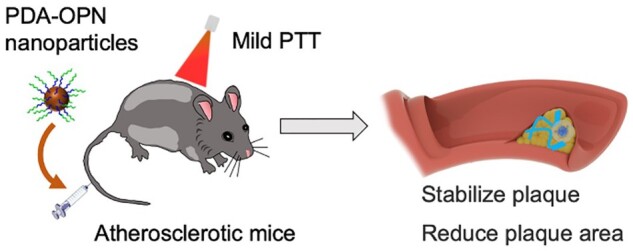

## Introduction

Atherosclerosis (AS) is a systemic chronic inflammatory disease characterized by plaque formation within the arterial wall, which is the pathological basis of cardiovascular diseases including myocardial infarction and cerebral infarction, posing a serious threat to human health [1, 2]. Taking lipid-lowering drugs is a routine clinical treatment for AS. However, due to the poor targetability of drugs resulting in low bioavailability, taking lipid-lowering drugs for the long term has limited efficacy in delaying AS and leads to side effects. Therefore, cardiovascular disease remains still the leading cause of death, which accounts for 32% of all deaths worldwide. It is worse that more than 23.6 million people will die of cardiovascular disease expectedly in 2030 [3]. Therefore, exploring new safe and efficient therapeutic methods for AS is crucial to slow down the disease and save patients' lives.

Disturbed lipid metabolism is a crucial factor in AS development, and foam cells play a vital role in this procedure. In the early stage of the disease, macrophages phagocytose large amounts of oxidized low-density lipoproteins (ox-LDL) and derive into foam cells. The dysregulated homeostasis of cholesterol uptake, storage and efflux leads to the aggregation of foam cells under the arterial wall. At the same time, macrophages-secreted pro-inflammatory cytokines drive foam cells and vascular smooth muscle cells to migrate and proliferate, forming plaque lipid nuclei and fibrous caps [4–6]. In the late stage of the lesion, many necrotic/apoptotic foam cells accumulate into the necrotic core, aggravating the inflammatory response and inducing plaque instability [7–10]. Therefore, modulation of lipid metabolism in foam cells is the ideal target for developing safe and efficient AS treatments.

With the advantages of good targetability, safety and controlled efficacy, nanotechnology-based treatments have become a hot area for anti-AS. Currently, nanoparticle-mediated photothermal therapy (PTT) has been applied to decline foam cells number and reduce the lipid content of plaques by photothermal ablation-induced cell apoptosis or necrosis [11]. Inorganic photothermal nanoparticles, such as gold, silica and copper sulfide, have made good progress in PTT of AS due to their high photothermal efficiency-induced cell apoptosis [12–17]. However, apoptosis and necrosis are independent hazard factors for AS lesions, and massive apoptosis or necrosis of foam cells can expand the necrotic core, reduce plaque stability and increase the risk of plaque rupture [18, 19]. Therefore, stabilizing plaque through reducing intracellular lipid levels but not inducing foam cell death remains a considerable challenge for developing safe and efficient PTT of AS. Very recently, we found that mild photothermal effect triggered by black titanium dioxide nanoparticles can significantly reduce lipid contents in foam cells via downregulating low-density lipoprotein receptor-dependent cholesterol uptake, increasing ATP-binding cassette transporter A1-mediated cholesterol efflux and inhibiting sterol regulatory element-binding protein 2-dependent cholesterol synthesis. Moreover, this mild photothermal effect can protect foam cells from apoptosis by upregulating the heat shock proteins [20]. However, such inorganic material cannot be metabolized *in vivo* and is concerned with potential long-term toxicity risk. Compared to inorganic materials, polydopamine (PDA) nanoparticles possess good biocompatibility and efficient photothermal capability, more importantly, can be quickly metabolized *in vivo* [21, 22]. Therefore, PDA nanoparticles have been widely introduced in cancer PTT but are still not reported in the field of anti-AS.

**Figure 1.**
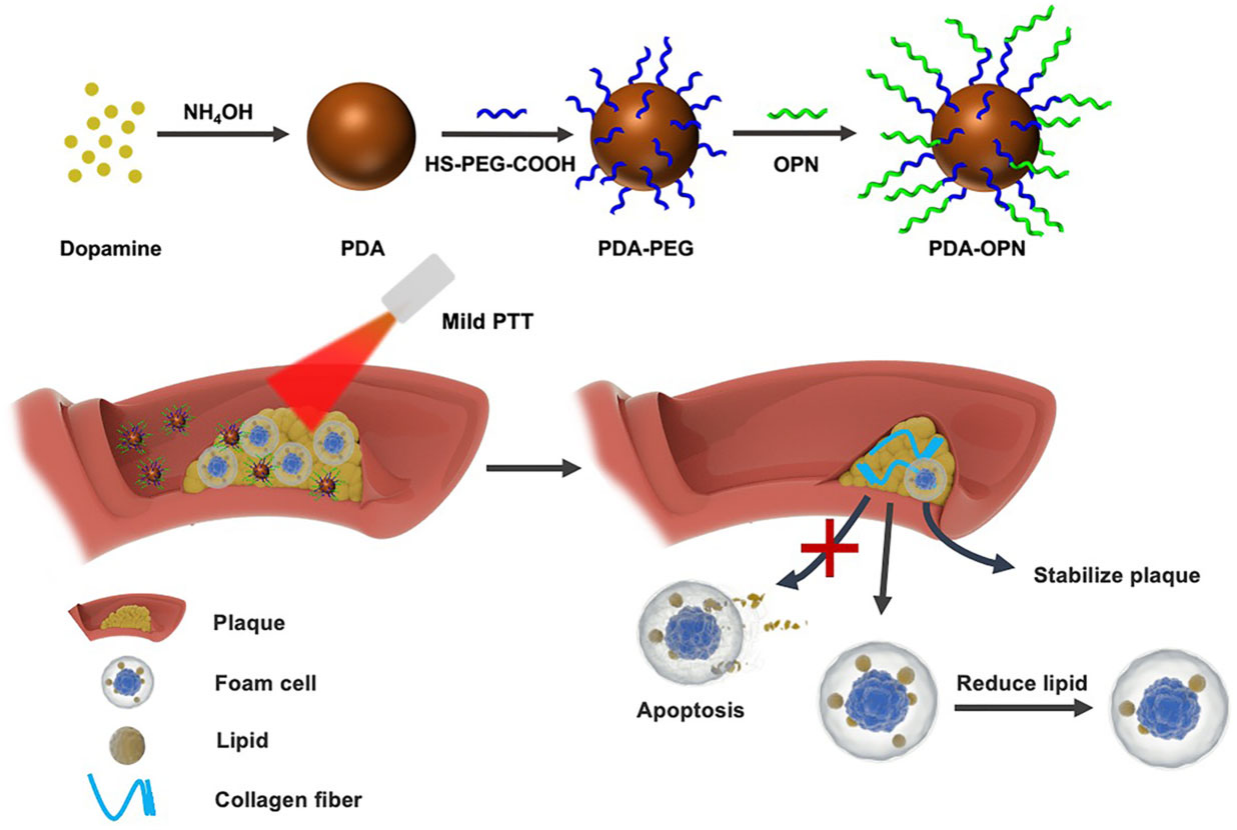
Schematic diagram of PDA-OPN nanoparticle-mediated mild PTT for treating atherosclerotic plaques.

This work first explored PDA nanoparticles for mild PTT of AS. Figure 1 illustrates a diagram of the study. PDA nanoparticles were modified with thiol-polyethylene glycol-carboxyl (HS-PEG-COOH) through the Michael reaction; then were coupled with active peptide-osteopontin (OPN) by the amido bond to form PDA-OPN nanoparticles. Notably, foam cells of atherosclerotic plaques highly express bone-bridging proteins [23, 24]. Therefore, after passing through the damaged endothelial cell gap, PDA-OPN nanoparticles can reach and accumulate in the plaque by specifically recognizing foam cells through their surface-modified active peptide-OPN. Under 808 nm laser irradiation, PDA-OPN-generated mild photothermal effect significantly reduces intracellular lipid. However, it does not induce cell apoptosis in foam cells. Subsequently, the PDA-OPN-triggered mild photothermal effect reduces plaque size and enhances collagenous fiber content, therefore improving plaque stability and inhibiting the development of AS in ApoE^−/−^ mice. Our study provides an alternative strategy for the safe and efficient therapy of AS.

## Materials and methods

### Materials

Dopamine hydrochloride was purchased from Macklin (Shanghai, China). Mercapto-polyethylene glycol-carboxyl (HS-PEG-COOH, 95%) was purchased from Yare (Shanghai, China). The active short peptide-OPN (90%) was purchased from Shanghai QiangYao Biotechnology Co. Ammonia solution (25–28%) and ethanol (99.5%) were purchased from Sinopharm (Shanghai, China).

### Synthesis of PDA-OPN nanoparticles

Four milliliters of ammonia solution (NH_4_OH, 25–28%), ethanol (40 ml) and deionized water (90 ml) were mixed and stirred for 30 min at 30°C. Then, 0.5 g of dopamine hydrochloride was added to 10 ml of water. After dissolving, the aqueous solution was mixed with the above mixture solution. The color of the solution quickly turned light brown and then gradually turned dark brown. After stirring for 16 h, the PDA nanoparticles were gained through centrifugation (12 000 rpm, 15 min) and washing with deionized water for three times.

Twenty-five milligrams of PDA and 50 mg of HS-PEG-COOH were then stirred in phosphate buffer saline (PBS) buffer at pH = 8.5 for 24 h. The suspension was centrifuged for 15 min at 12 000 rpm to obtain PDA-PEG nanoparticles. Finally, PDA-PEG (10 mg), 1-ethyl-3-(3-dimethylaminopropyl) carbodiimide (2.2 mg) and N-hydroxysuccinimide (1 mg) were dissolved in deionized water and magnetically stirred for 4 h at room temperature. After that, 15 mg OPN aqueous solution (1 ml) was added and stirred for 16 h. The PDA-OPN nanoparticles were recovered after centrifugation for 15 min at 12 000 rpm.

### Characterization

The morphology of PDA, PDA-PEG and PDA-OPN nanoparticles was performed by transmission electron microscopy (TEM, Tecnai F30, USA). A dynamic light scattering analyzer (Nano ZS, England) was applied to analyze the diameter and zeta potential of the nanoparticles. The ultraviolet-visible (UV-vis) absorption spectra were obtained from a UV-vis spectrophotometer (T10CS, China).

### Photothermal performance of PDA-OPN

To test the photothermal performance of PDA-OPN, 1.0 ml of PDA-OPN in aqueous solution with various concentrations (50, 100 and 150 μg⋅ml^−1^) were held in dishes respectively and then were for 10 min irradiation at 1.0 W⋅cm^−2^ by 808 nm laser. The temperatures and thermographic images were performed with the infrared thermal imaging instrument.

### Cell culture and macrophage-derived foam cell formation

RAW 264.7 cells were cultured at 37°C in a 5% CO_2_ humidified environment and were provided dulbecco's modified eagle medium (DMEM) supplemented with 10% fetal bovine serum (FBS) and 1% antibiotics. RAW 264.7 cells were stimulated with 50 μg⋅ml^−1^ ox-LDL solution prepared from DMEM medium containing 5% FBS for 48 h, resulting in the formation of foam cells.

### Cell viability assay

The cytotoxicity of PDA-PEG and PDA-OPN nanoparticles were assayed on macrophage-derived foam cells. Cells were inoculated at 2 × 10^4^ cells per well in 96-well plates and incubated overnight in the incubator (37°C, 5% CO_2_). Then, PDA-PEG and PDA-OPN nanoparticles in various concentrations (0, 6.25, 12.5, 25, 50 and 100 μg⋅ml^−1^) were added to further incubate the cells for 24 h. After that, cell viability was detected using Cell Counting Kit-8 (CCK-8) assay.

### Cellular uptake

Rhodamine was used for labeling the nanoparticles to trace the intracellular distribution of the PDA-PEG and PDA-OPN nanoparticles. Briefly, rhodamine was added to an aqueous solution of nanoparticles. After stirring for 24 h under dark conditions, the solution was washed and centrifuged to receive rhodamine-labeled PDA-PEG or PDA-OPN nanoparticles. Foam cells were treated for 4 h with medium containing 100 μg⋅ml^−1^ of PDA-PEG or PDA-OPN nanoparticles. Then, the cells were stained by Hoechst with light avoidance and observed from confocal laser scanning microscopy (CLSM). The excitation wavelengths were 405 and 552 nm, and the emission wavelengths were 415–480 and 600–700 nm.

### *In vitro* mild phototherapy

Foam cells were inoculated at 3 × 10^4^ cells/well in 96-well plates and incubated overnight. After that, 100 μg⋅ml^−1^ of PDA-PEG and PDA-OPN nanoparticles containing culture solution were added into cells, and then incubated for 4 h. The cells were irradiated for 10 min (808 nm, 1.0 W⋅cm^−2^). After another incubation overnight, cell viability was obtained through CCK-8 assay.

### Live/dead cell staining

After mild photothermal treatment, the cells were stained by Calcein-AM/PI solution for 0.5 h under dark conditions and then were observed from fluorescence microscopy. Live cells presented green fluorescence, and dead cells showed red staining. The excitation wavelengths were 470 and 560 nm, respectively.

### Foam cell formation assay

The treated cells were soaked in 4% paraformaldehyde for 0.5 h, infiltrated for 5 min with 60% isopropanol and then incubated for 1 h with Oil Red O staining solution. A light microscopy was performed to observe the cells, and ImageJ software was used to calculate the percentage of intracellular lipid droplet area.

### Animal model

ApoE^−/−^ mice (6 weeks old, male) were purchased from Nanjing Qingzilan Technology Co. The mice were fed for 12 weeks with a high-fat diet (4% milk powder, 10% lard, 1.5% cholesterol and 0.5% sodium cholate) to establish an AS model and placed in an animal room at suitable temperature with a dark/light cycle of 12 h.

### *In vivo* anti-atherosclerotic effect

Atherosclerotic mice were grouped randomly: (i) AS+PBS; (ii) AS+PDA-PEG; (iii) AS+PDA-PEG+Laser; (iv) AS+PDA-OPN; and (v) AS+PDA-OPN+Laser. After 8 h of injection via tail vein, the aortic region of each group was treated with irradiation (808 nm, 10 min, 1.0 W⋅cm^−2^) at 3-day intervals for a treatment cycle of 14 days.

During mild PTT, the temperature change of the aortic lesion site in mice was monitored using an infrared thermal imaging instrument. After treatment, the entire aorta was stripped down, the perivascular tissue was excised, and the aorta was cut longitudinally. Atherosclerotic plaque in the aortas was stained by Oil Red O staining solution for 15 min, and the aortas were cleaned with PBS after staining. Subsequently, the whole aorta was unfolded and flattened to fully expose the intima, and the aortic plaque was observed using a light microscope and photographed. In addition, the aortic root was sectioned in cross-section and used for histological analysis through hematoxylin–eosin (H&E) staining, and the lipid content of plaque area was detected using Oil Red O staining. Masson staining was used to evaluate the content of collagen fiber in the plaque area. The percentage of plaque and collagen fiber areas was calculated by ImageJ software.

### *In vivo* biocompatibility

Healthy mice were injected with PBS, PDA-PEG and PDA-OPN nanoparticles via tail vein. Body weight was recorded within 14 days, blood was collected for hematological analysis (routine blood, blood biochemistry), and organs (heart, lung, kidney, spleen and liver) were taken for H&E staining after treatment to assess the toxicity of nanoparticles jointly.

### Statistical analysis

All data were expressed as mean±standard deviation. Statistics analysis was performed by GraphPad Prism 9 (GraphPad Software, USA) with the one-way analysis of variance and the two-tailed, unpaired t-test. A *P* < 0.05 was assessed as statistical significance.

## Results and discussion

### Characterization of nanoparticles

**Figure 2.**
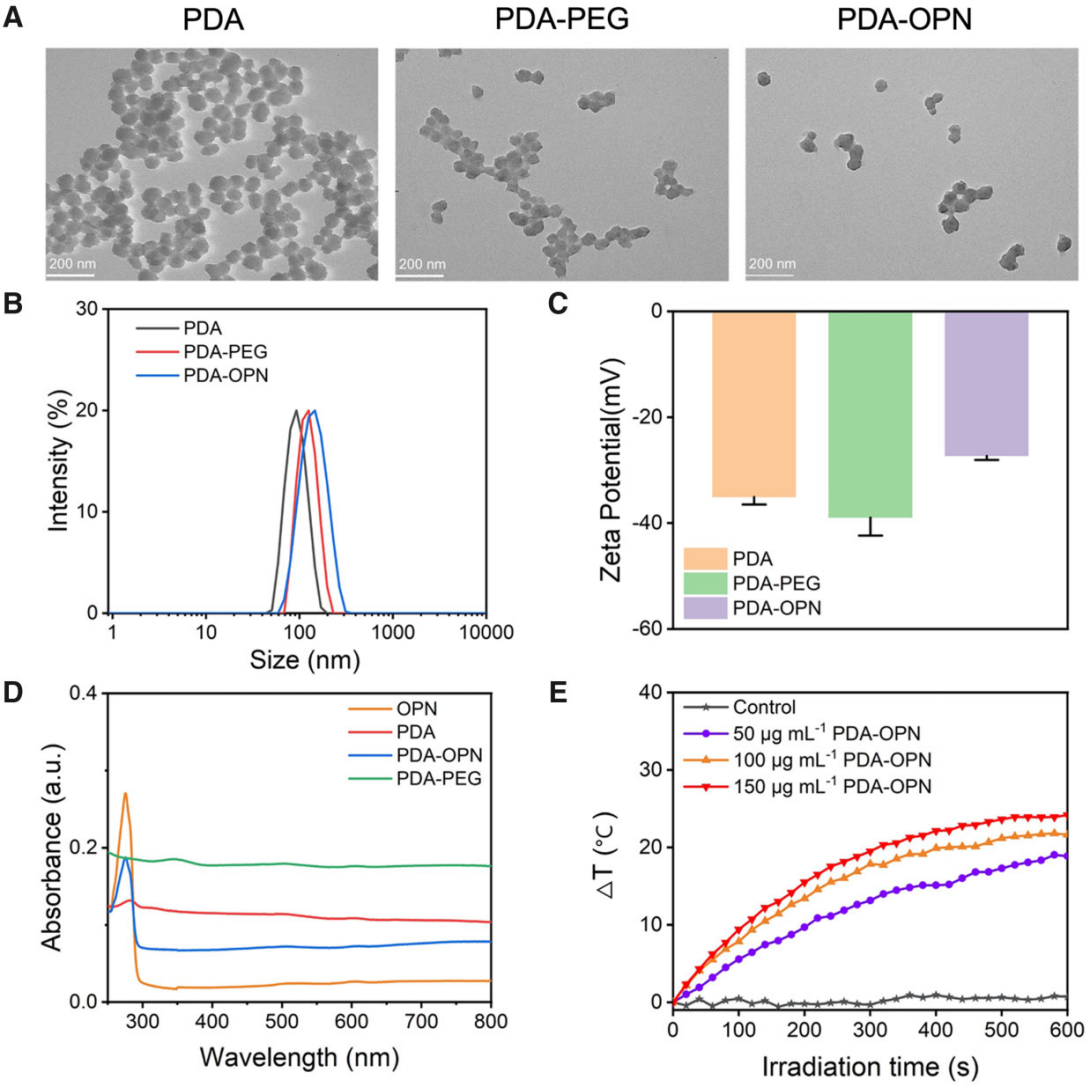
(**A**) TEM images of PDA, PDA-PEG and PDA-OPN, and the corresponding (**B**) hydrodynamic diameters, (**C**) zeta potential and (**D**) UV-vis absorbance spectra. (**E**) Temperature curves of PDA-OPN in various concentrations irradiated by 1.0 W⋅cm^−2^ of 808 nm laser.

As the TEM images shown in Fig. 2A, the sizes of PDA, PDA-PEG and PDA-OPN nanoparticles are 70, 80 and 90 nm, respectively. It can be seen from Fig. 2B that the hydrodynamic diameters of PDA, PDA-PEG and PDA-OPN nanoparticles are 93, 130 and 142 nm, respectively. After modifying with PEG and OPN, the hydrodynamic size increases accordingly, which proves that PEG and OPN are successfully modified on PDA nanoparticles. The polydispersity index of PDA, PDA-PEG and PDA-OPN nanoparticles are 0.10, 0.23 and 0.18, respectively, indicating that the nanoparticles have good dispersibility in liquid solution. Figure 2C exhibits that the surface charge of PDA nanoparticles is −35 ± 1.4 mV. Due to PEG containing a large number of hydroxyl groups, the surface charge of PDA-PEG is −39 ± 3.4 mV. After conjugating with OPN through amide linkage between the carboxyl and amino groups, the surface charge of PDA-OPN increases to −27 ± 0.8 mV but still indicates good colloidal stability. The UV-vis absorption spectra (Fig. 2D) show no distinction in the change of PDA and PDA-PEG nanoparticles, indicating that PEG does not affect the absorption of PDA. OPN has a clear proteic absorption peak at 275 nm, and PDA-OPN has the same absorption peak at 275 nm, meaning that OPN was successfully coupled with PDA-PEG.

To assess the photothermal performance of PDA-OPN, 50–150 μg⋅ml^−1^ of nanoparticles were dispersed in pure water and subsequently irradiated by 808 nm laser (10 min, 1.0 W⋅cm^−2^). Figure 2E shows that the PDA-OPN nanoparticles' temperature reaches 43.7, 46.4 and 48.5°C, corresponding to the concentrations of 50, 100 and 150 μg⋅ml^−1^. In contrast, with the same conditions, the deionized water temperature only increases by 0.7°C. Consideration of mild photothermal requirements, 100 μg⋅ml^−1^ of PDA-OPN could be an optional concentration for the following cellular and animal study.

### Cell-targeting capacity of PDA-OPN nanoparticles

**Figure 3.**
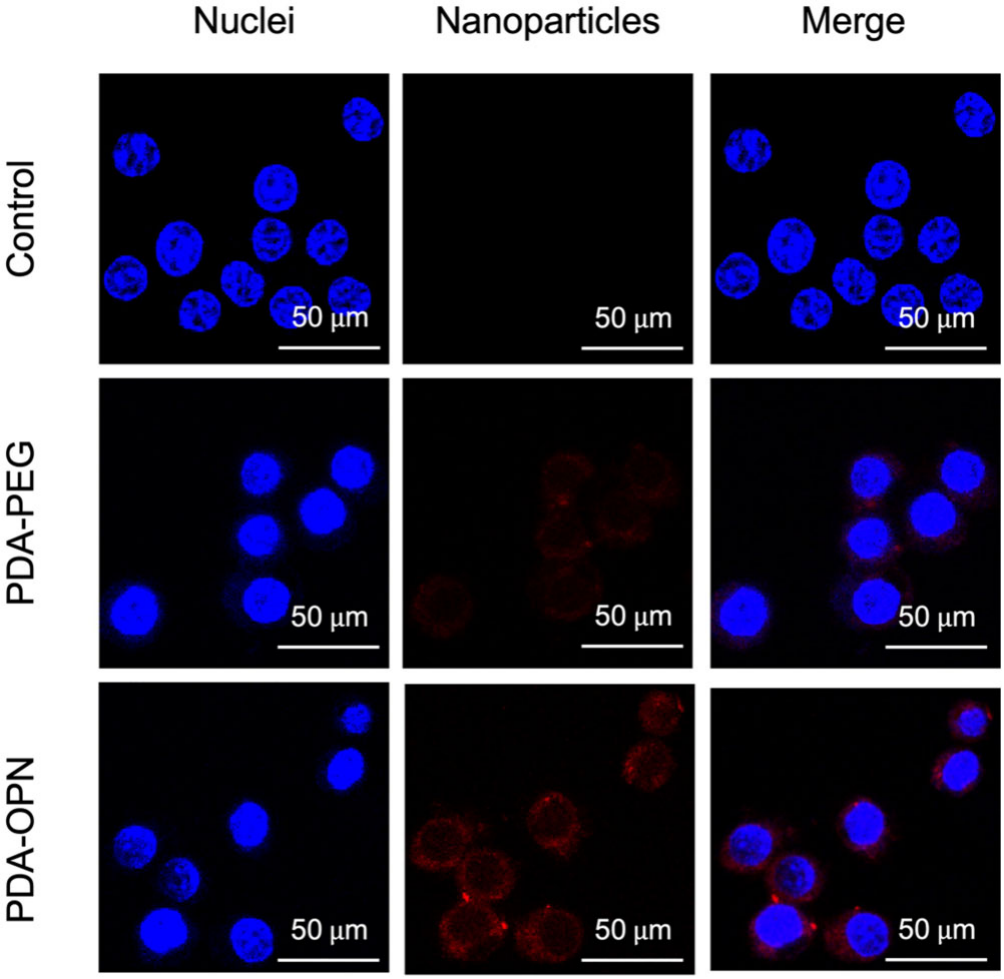
CLSM images of foam cells treated with or without rhodamine-labeled PDA-PEG or PDA-OPN nanoparticles for 4 h. Scale bar: 50 μm.

Since foam cells highly express OPN, a short stretch of active peptide chain was selected as a ligand for targeting OPN. The cell-targeting capacity of PDA-OPN nanoparticles was determined by cellular uptake analysis. As shown in Fig. 3, there are no apparent changes in nuclei morphology before and after co-incubation with nanoparticles. The red fluorescence indicates that the PDA-PEG and PDA-OPN nanoparticles mainly distribute in the cytoplasm after entering foam cells. Among them, the highest red fluorescence accumulation and intensity are observed in the PDA-OPN group, proving that the PDA-OPN nanoparticles have an excellent targeting ability to the foam cells, thus benefit for the subsequent therapeutic effect.

### Mild PTT in foam cells

**Figure 4.**
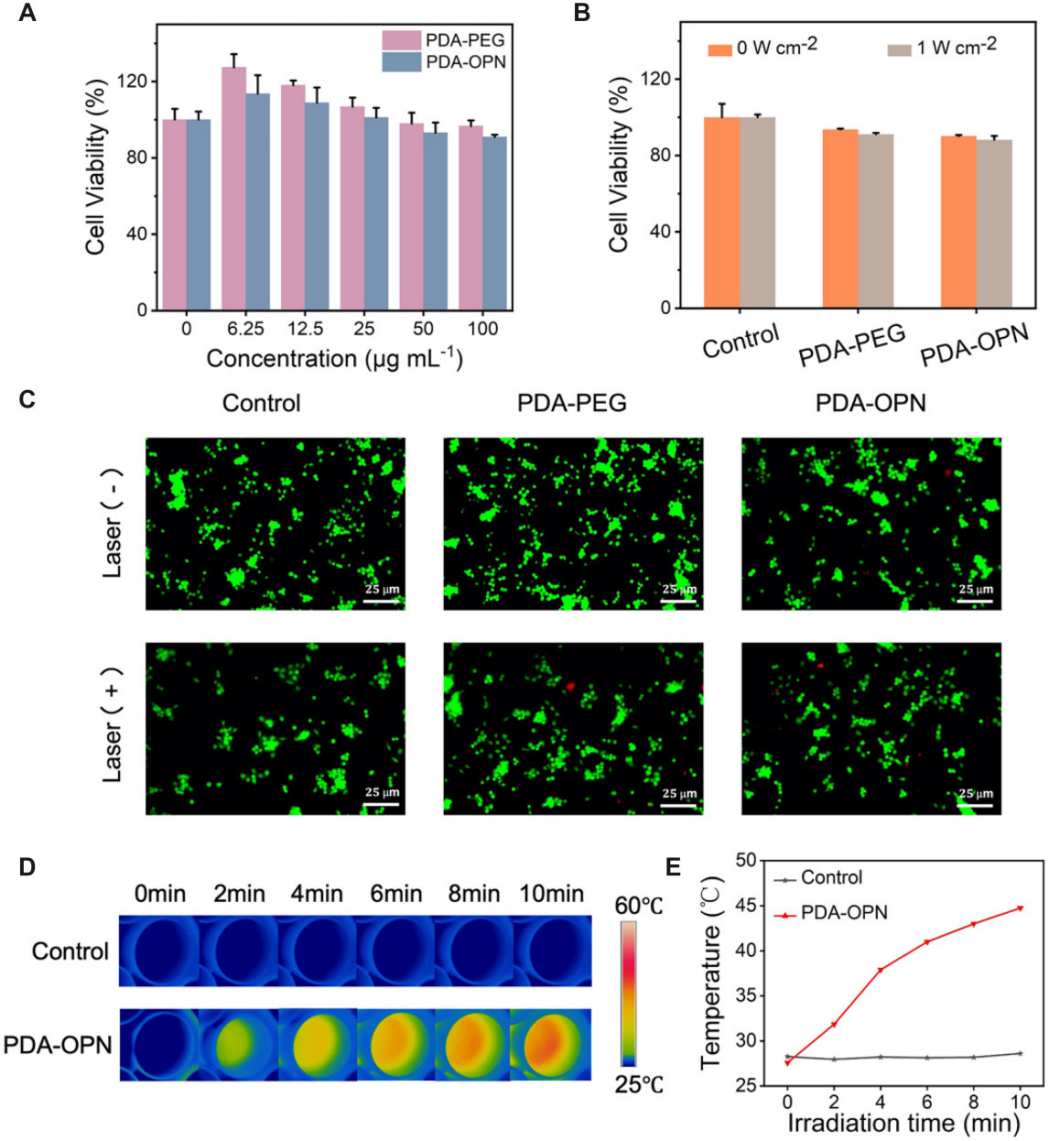
(**A**) Cell viabilities of foam cells treated with different samples and concentrations, and (**B**) irradiated with or without 808 nm laser (1.0 W⋅cm^−2^, 10 min, 100 μg⋅ml^−1^). (**C**) Fluorescence live/dead cell images of foam cells after different treatments. Scale bar: 25 μm. (**D**) Infrared thermal images of foam cells after treatment with PDA-OPN (100 μg⋅ml^−1^) and irradiation for 10 min (808 nm, 1.0 W⋅cm^−2^) and (**E**) corresponding temperature curves.

Before the cell treatment, we assessed the cytotoxicity of PDA-PEG and PDA-OPN nanoparticles to determine the optimum concentration. Foam cells were incubated with different concentrations of PDA-PEG and PDA-OPN for 24 h. The results (Fig. 4A) of CCK-8 experiments show that 100 μg⋅ml^−1^ of PDA-OPN has no apparent effect on foam cell viability. Combination of the photothermal curve obtained from Fig. 2E, 100 μg⋅ml^−1^ of nanoparticles were selected to carry out the following therapeutic experiments.

Currently, PTT is mainly used to inhibit the progression of AS by inducing cell apoptosis and necrosis to reduce the number of foam cells and lower the lipid level of plaques. However, excessive apoptosis and necrosis can evolve into a necrotic core, exacerbating the inflammatory response and reducing plaque stability, thereby increasing the risk of plaque rupture [11, 20]. Therefore, avoiding foam cell apoptosis while regulating cellular lipid metabolism is an urgent challenge to solve for AS's PTT. As shown in Fig. 4B, the cell viability in PDA-OPN group remains above 88% after 808 nm laser irradiation. The live–dead co-staining was performed to visualize the cell survival status under mild PTT (Fig. 4C). Consistent with the CCK-8 results, PDA-OPN nanoparticle-mediated mild PTT does not cause significant cell death. After co-incubating PDA-OPN nanoparticles with foam cells, we monitored the intracellular temperature changes by an infrared thermal imaging instrument (Fig. 4D and E). The temperature of the PDA-OPN group is increased from 27.6 to 44.7°C with irradiation (808 nm, 1.0 W⋅cm^−2^, 10 min). These results are consistent with our recent study of inorganic nanoparticle-based mild PTT of AS, which finds foam cells can express heat shock protein under mild photothermal below 45°C, which protects the cell against apoptosis [20].

**Figure 5.**
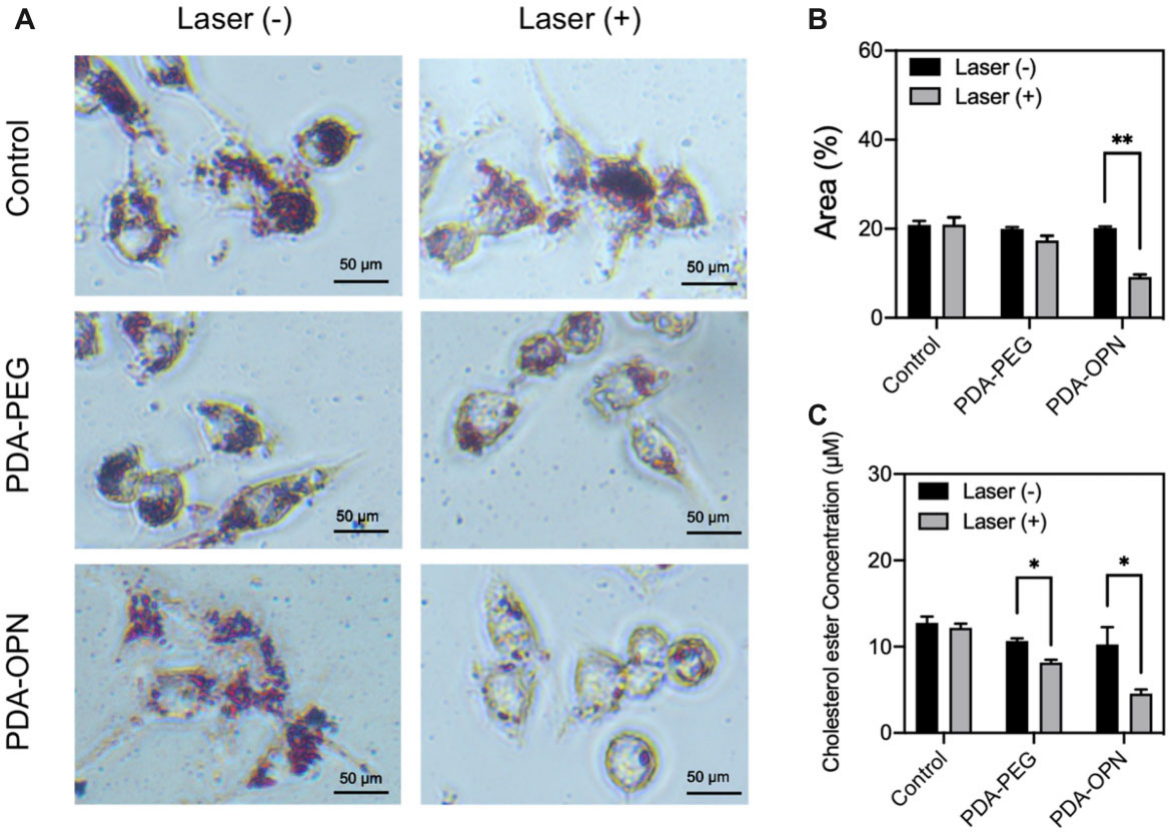
Regulation of mild PTT on intracellular lipid metabolism. (**A**) Representative images of foam cells stained with oil red O and (**B**) quantified lipid content in foam cells (*n* = 3). Scale bar: 50 μm. (**C**) Cholesterol ester concentration in foam cells under different treatment conditions (*n* = 3). *P < 0.05, **P < 0.01.

To investigate nanoparticle-mediated moderation of lipid metabolism by mild phototherapy, we studied the lipid accumulation in foam cells treated with PDA-PEG and PDA-OPN with or without irradiation by 808 nm laser. As shown in Fig. 5A and B, the lipid in foam cells is revealed by Oil red O staining. After irradiation, the lipid content in PDA-OPN group dramatically decreases from 20.2% to 9.2% (*P* < 0.01). For further study, we examined the intracellular cholesteryl ester content in different treatments (Fig. 5C). The results show that the cholesteryl ester content in PDA-OPN group is reduced from 10.3 to 4.6 µM (*P* < 0.05) after irradiation, which is consistent with the Oil red O staining results. Therefore, these results suggest that PDA-OPN-mediated mild PTT can regulate cellular lipid metabolism without causing evident apoptosis or necrosis.

### Photothermal therapy *in vivo*

**Figure 6.**
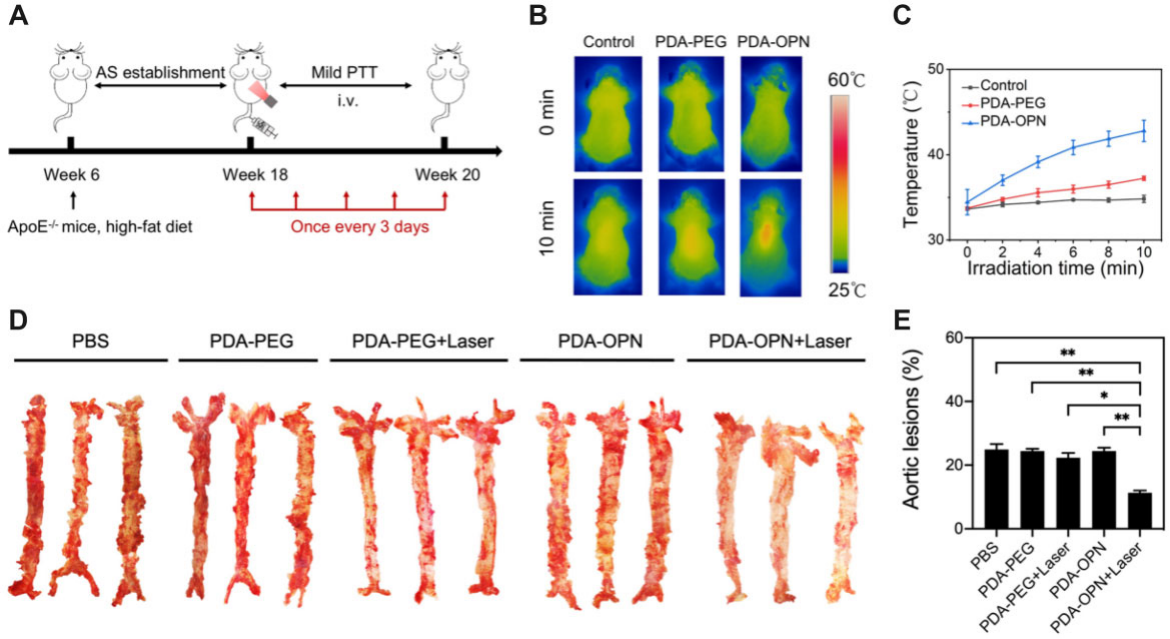
(**A**) Diagram of the treatment process of atherosclerotic mice. (**B**) Infrared thermal images of the aorta under irradiation. (**C**) Temperature-elevating curves of the aorta with 808 nm laser irradiation (*n* = 3). (**D**) Representative images of the whole aorta stained by Oil Red O staining in atherosclerotic mice with different treatments. (**E**) Quantitative analysis of aortic lesion area (*n* = 3), *P < 0.05, **P < 0.01.

Next, we evaluated the *in vivo* therapeutic effect of PDA-OPN nanoparticle-mediated mild PTT on AS. The animal experiments of this study strictly observed the guideline of the Experimental Animal Ethics Committee of Ningbo University (Permit No. SYXK Zhe 2019-0005). As shown in Fig. 6A, ApoE^−/−^ mice in 6-week-old were fed for 12 weeks with a high-fat diet to form atherosclerotic plaques. Then they were grouped randomly and given different treatments: PBS group, PDA-PEG group, PDA-PEG + Laser group, PDA-OPN group and PDA-OPN + Laser group, respectively. All mice were injected through the tail vein with PBS or nanoparticles and treated every 3 days for 14 days. A high-fat diet was fed simultaneously during the treatment period. After treatment, the mice were executed to assess the treatment effect. Eight hours after injection, we monitored the temperature change at the lesion site in mice by infrared thermal imaging instrument. Figure 6B and C indicates that the temperature of the aortic site in the PDA-OPN group increases from 34.4 to 42.8°C after irradiation by 808 nm laser (1.0 W⋅cm^−2^, 10 min). Studies have shown that nanoparticles can pass through the damaged endothelial gap and reach the subendothelial layer after entering the blood circulation [25]. Therefore, when PDA-OPN nanoparticles reach subendothelial layer, they can specifically recognize foam cells through their surface-modified active peptide-OPN and accumulate in the plaque. As shown in Fig. 6D and E, three aortas in each group were taken for Oil red O staining, and the area in red indicates the atherosclerotic plaques. It is clearly displayed that the number of plaques is decreased in the PDA-OPN + Laser group when compared with other groups. Oil Red O staining images results show that the aortic plaque area is significantly reduced in the PDA-OPN group under irradiation. The quantification results show that the plaque area in the PBS, PDA-PEG, PDA-PEG + Laser and PDA-OPN groups is 24.9%, 24.4%, 22.3% and 24.4%, respectively, while the plaque area is reduced to 11.3% in the PDA-OPN + Laser group. The above results demonstrate that PDA-OPN nanoparticle-mediated mild PTT can specifically decrease lipid content in plaques.

**Figure 7.**
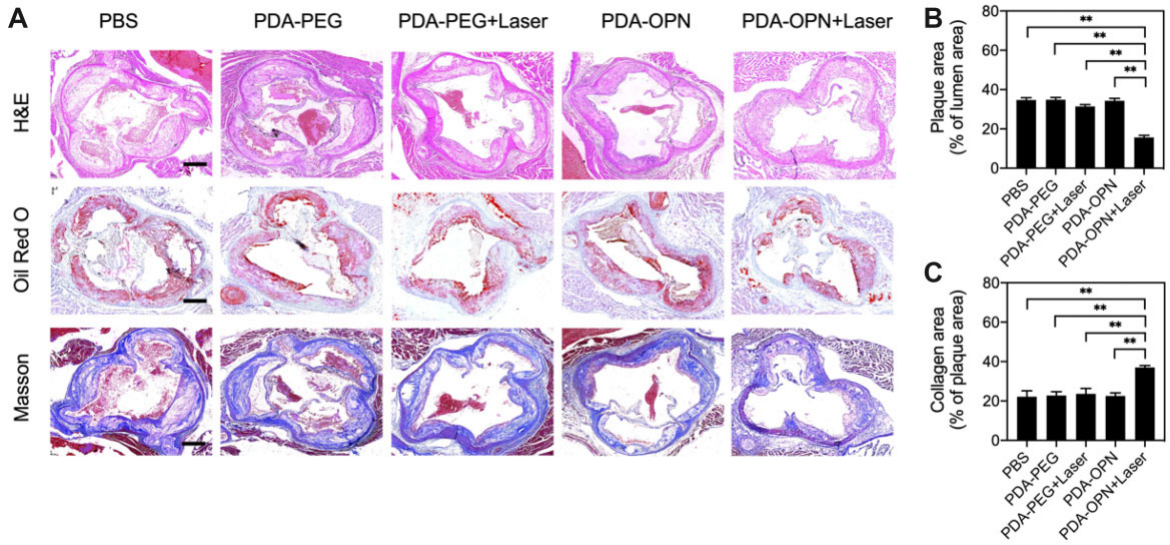
(**A**) Representative images of staining (H&E, Oil red O and Masson’s trichrome) the aortic root cross-sections from ApoE^−/−^ mice following various treatments. Scale bar: 250 μm. (**B**) Quantitative analysis of plaque area expressed as the percentage of Oil red O positive area in the aortic root lumen area (*n* = 3). (**C**) Quantitative analysis of collagen area expressed as the percentage of collagen-positive area in plaque area (*n* = 3). **P < 0.01.

The treatment effect was further verified by histological analysis of aortic arch cross-sections. As shown in Fig. 7A, the H&E and Oil red O staining results show a larger plaque area and lipid enrichment in control groups. However, the plaque area and lipid content are significantly reduced in the PDA-OPN + Laser group. The quantification results confirm that the plaque area in the PBS, PDA-PEG, PDA-PEG + Laser and PDA-OPN groups is 34.7%, 34.8%, 31.4% and 34.3%, respectively, while mild PTT mediated by PDA-OPN nanoparticles could reduce the plaque area to 15.6% (Fig. 7B). As shown in Fig. 7C, Masson trichrome staining suggests that the PDA-OPN nanoparticles-mediated mild PTT exhibits enhanced plaque fibrosis compared with control groups. The collagen area in the PBS, PDA-PEG, PDA-PEG + Laser and PDA-OPN groups is 22.2%, 22.8%, 23.6% and 22.6%, respectively, while mild PTT mediated by PDA-OPN nanoparticles could increase the collagen area to 37.0%. Atherosclerotic plaques rich in lipid necrotic nuclei and low in fiber content are unstable and prone to form vulnerable plaques that can easily rupture and cause acute cardiovascular events [2]. Therefore, downregulating cellular lipids and upregulating fiber content can improve the stability of plaque. The above staining results further validate that PDA-OPN nanoparticles-mediated mild PTT can effectively inhibit AS and stabilize plaques.

### *In vivo* biocompatibility

To evaluate the nanoparticle's *in vivo* toxicity, healthy mice were injected with PBS, PDA-PEG, and PDA-OPN nanoparticles via tail vein (10 mg⋅kg^−1^). Blood was collected for blood routine and biochemical tests after 14 days of treatment, and organs (heart, lung, kidney, spleen and liver) were stained by H&E. There is no obvious change in body weight (Supplementary Fig. S1), routine blood (Supplementary Fig. S2) and biochemical blood parameters (Supplementary Fig. S3) between the three groups. Meanwhile, the H&E staining results (Supplementary Fig. S4) also suggest no pathological or inflammatory injury in the main organs, indicating that PDA-OPN nanoparticles have good biocompatibility for mice under the injected dose and observing time.

## Conclusions

In summary, we report the potential of PDA-OPN nanoparticle-mediated mild PTT for treating AS. The synthesized PDA-OPN nanoparticles exhibit good photothermal effects and targeting properties against macrophage-derived foam cells. Importantly, unlike conventional PTT, the mild temperature generated by PDA-OPN nanoparticles does not cause massive cell death, dramatically reducing vascular injury due to apoptosis and necrosis. On the other hand, the PDA-OPN nanoparticles can regulate the lipid metabolism of foam cells in plaque, significantly reduce intracellular lipid accumulation, and effectively reduce plaque area. In addition, PDA-OPN nanoparticle-mediated mild PTT can significantly enhance plaque fiber contents, which improves plaque stability and inhibits AS's progress. Therefore, this study provides a new strategy for developing mild PTT-based safe and efficient AS treatment.

## Supplementary Material

rbad031_Supplementary_Data
