# Supplementary material for: Polydopamine nanoparticle-mediated mild photothermal therapy for inhibiting atherosclerotic plaque progression by regulating lipid metabolism of foam cells
Source: Regen Biomater. 2023 Mar 25;10:rbad031. doi: 10.1093/rb/rbad031 (PMC10081882; doi:10.1093/rb/rbad031)
Supplement: rbad031_Supplementary_Data [file rbad031_supplementary_data.docx]

**Polydopamine Nanoparticle-mediated Mild Photothermal Therapy for Inhibiting Atherosclerotic Plaque Progression by Regulating Lipid Metabolism of Foam Cells**

Shuangshuang Tu^1,2^, Wenzhi Ren^2,4*^, Jinru Han^2,3^, Haijing Cui^2^, Ting Dai^1^, Haoxuan Lu^1^, Yanqing Xie^1^, Wenming He^1*^ and Aiguo Wu^2,4*^

^1^ Department of Cardiology, The First Affiliated Hospital of Ningbo University, 247 Renmin Road, Jiangbei District, Ningbo, Zhejiang Province, 315020, China

^2^ Cixi Institute of Biomedical Engineering, International Cooperation Base of Biomedical Materials Technology and Application, Chinese Academy of Science (CAS) Key Laboratory of Magnetic Materials and Devices & Zhejiang Engineering Research Center for Biomedical Materials, Ningbo Institute of Materials Technology and Engineering, CAS, 1219 ZhongGuan West Road, Ningbo 315201, China

^3^ University of Chinese Academy of Sciences, No. 1 Yanqihu East Road, Huairou District, Beijing, 101408, China

^4^ Advanced Energy Science and Technology Guangdong Laboratory, Huizhou, 516000, China

^*^ Corresponding author. E-mail: renwzh@nimte.ac.cn, fyhewenming@nbu.edu.cn, aiguo@nimte.ac.cn


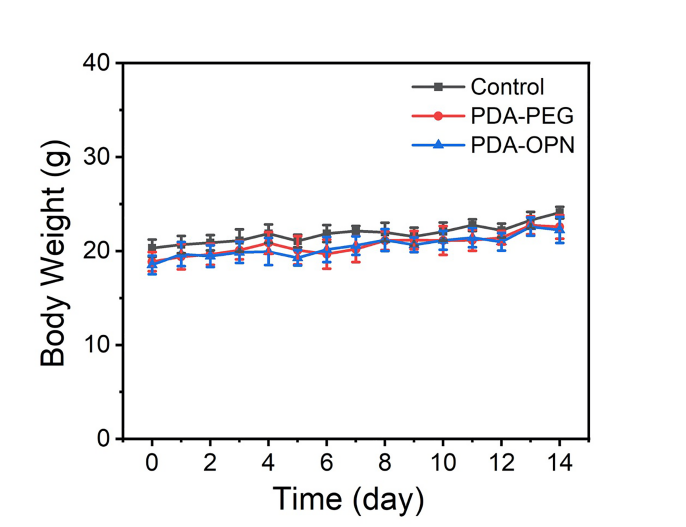


**Fig. S1** Body weight changes during the 14-d treatment observation period (n = 5).


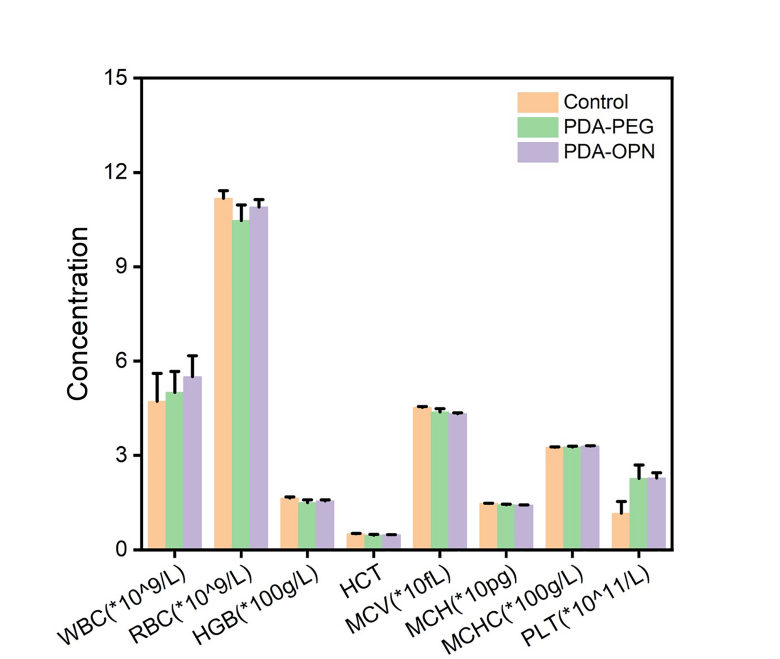


**Fig. S2** Blood routine examination including WBC, RBC, HGB, HCT, MCV, MCH, MCHC, and PLT of mice with or without treatment (n = 3). WBC: white blood cell. RBC: red blood cell. HBG: hemoglobin. HCT: hematocrit. MCV: mean corpuscular volume. MCH: mean corpuscular hemoglobin. MCHC: mean corpuscular hemoglobin to determine blood drug concentration. PLT: platelet.


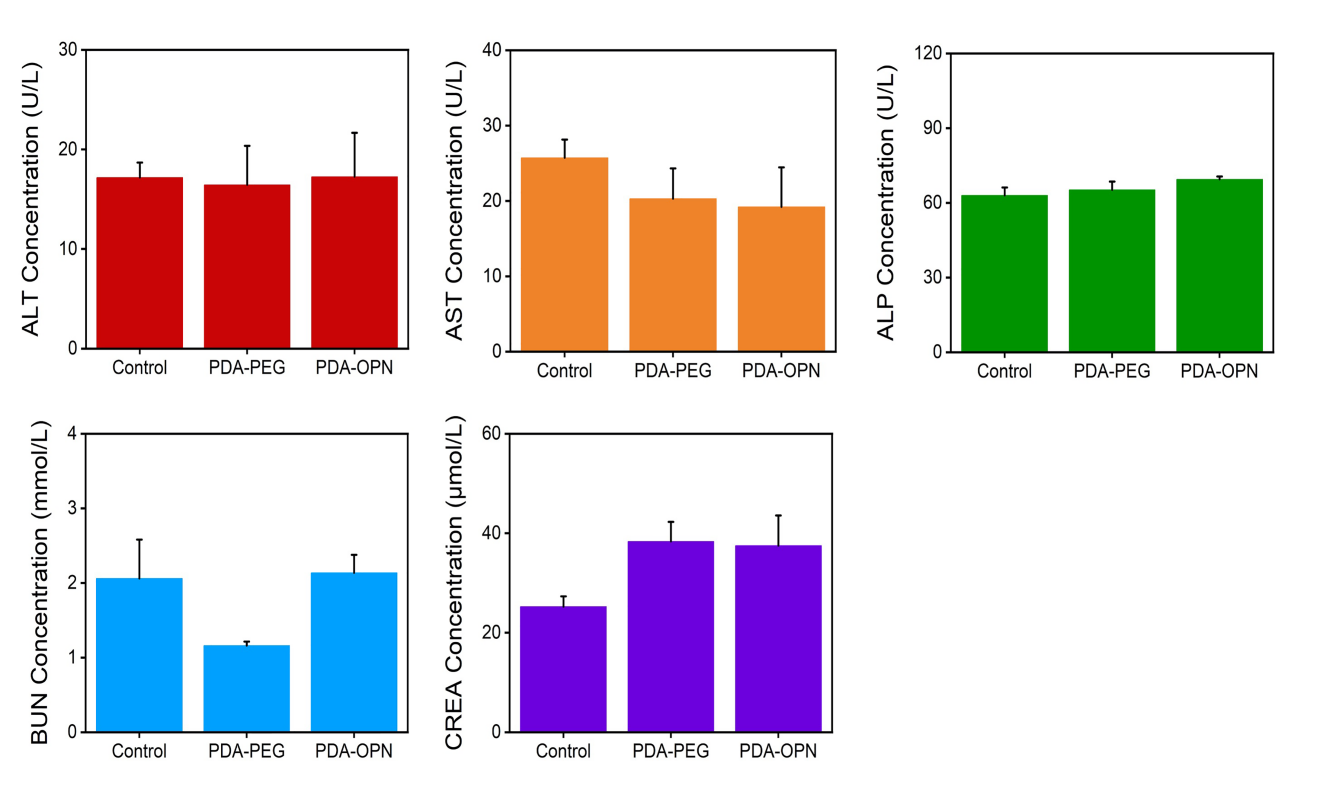


**Fig. S3** The serum levels of liver function indicators including alanine transaminase (ALT), aspartate transaminase (AST), and alkaline phosphatase (ALP), and kidney function indicators involving blood urea nitrogen (BUN) and creatinine (CREA) of different groups (n = 3).


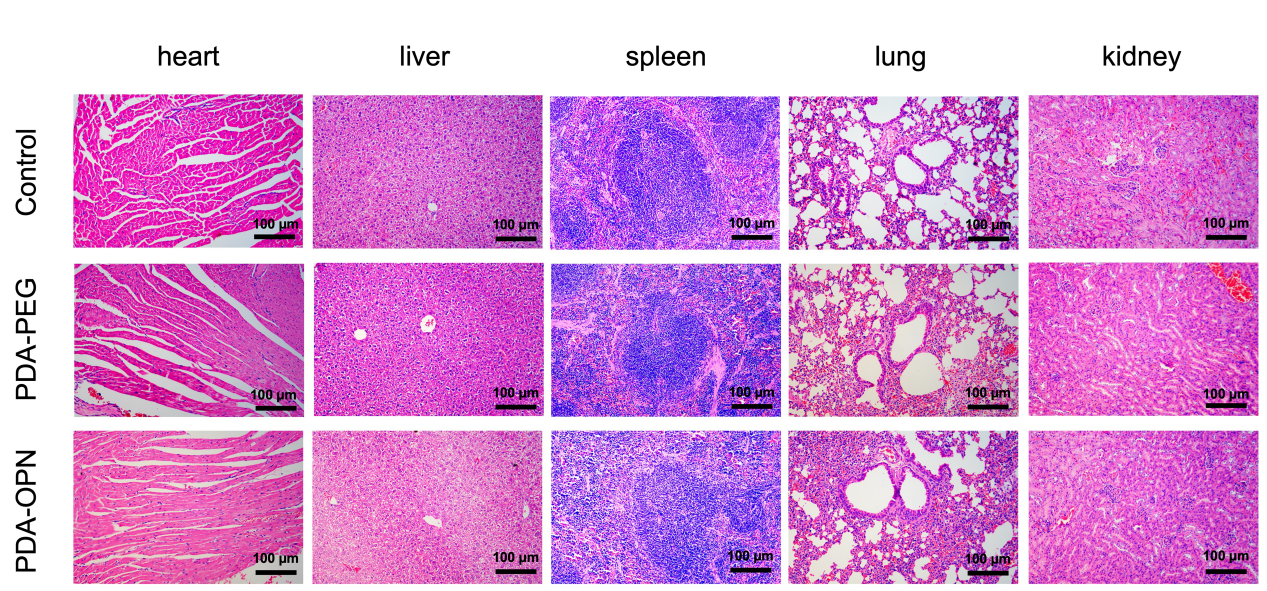


**Fig. S4** H&E staining images of major organs (heart, liver, spleen, lung, and kidney) of different groups. Scale bar: 100 μm.
